# Applications of artificial intelligence in endodontic education: a systematic review

**DOI:** 10.3389/fdmed.2026.1844279

**Published:** 2026-07-14

**Authors:** Mohammed Mustafa, Ahmed A. Almokhatieb, Abdulaziz Abdulwahed, Laila S. Almufleh, Shahad Albader, Mohsin Bilal

**Affiliations:** 1 Department of Conservative Dental Sciences, College of Dentistry, Prince Sattam Bin Abdulaziz University, Al-Kharj, Saudi Arabia; 2 Information Systems Department, College of Computer Engineering and Sciences, Prince Sattam Bin Abdulaziz University, Al-Kharj, Saudi Arabia

**Keywords:** artificial intelligence, dental education, endodontics, machine learning, virtual reality

## Abstract

**Background:**

The growing bases of Artificial Intelligence (AI) applications ranging from diagnostic support to immersive training have rapidly advanced in the dental education field. Endodontics, by its very nature of relying so highly on a proper diagnosis and careful technical execution, is an indication through which AI may be best poised to succeed the most in specialty care.

**Objective:**

The aim of this systematic review was to assess the role of artificial intelligence (AI): machine learning (ML), deep learning (DL), virtual/augmented reality (VR/AR) and large language models (LLMs) related to endodontic education based on available evidence published until September 2025.

**Methods:**

This review was performed in accordance with the PRISMA 2020 guidelines. Publication databases were reviewed included PubMed, Scopus, Web of Science and Cochrane. Inclusion Criteria: Studies that evaluated any form of AI for didactic, preclinical or clinical education in endodontics and/or patient-centered education were included. Study characteristics, AI domains, applications and outcomes were extracted. Risk of bias and methodological quality were evaluated according to study design using RoB 2, ROBINS-I, AXIS, and AMSTAR-2 tools.

**Results:**

Fifteen studies were included. Radiographic interpretation augmented by AI improved sensitivity and specificity to reduce false positive reporting especially for junior clinicians. In preclinical training, VR/AR simulations have shown to improve psychomotor skills, confidence and knowledge acquisition. LLMs can be useful in producing exam questions and case-based Q&A, although the accuracy and discriminatory ability varied. AI mediated Patient education interventions led to anxiety reduction and comprehension. There was heterogeneity of outcome measures, dataset bias; reliability and transparency issues.

**Conclusion:**

AI holds promise for use in diagnostic, didactic and preclinical endodontic education. They must be safely implemented in a controlled format, under the supervision of faculty and with objective evaluation metrics in place.

**Clinical significance:**

AI provides quantifiable benefits in endodontic education by improving accuracy of diagnosis, assisting decision-making and facilitating dental students training using VR/AR simulation. Some interventions using AI in curricula may allow the student to acquire skills faster, feel more confident, and transfer these benefits to improved patient communication. But we need to make sure our integration is backed up with faculty monitoring, transparent AI models and rigorous validation before putting it in any production environment or relying on it too heavily for exam outcomes.

## 1 Introduction

Over the last 10 years, there has been an explosion of artificial intelligence (AI) in health care—with regard to breadth and scale of AI-based solutions applied, dentistry is one of the most active fields. AI encompasses ML and DL tools like computer vision and NLP capable of recognizing patterns within terabytes of data (e.g., electronic health records) as well as producing clinically meaningful outputs with limited human in the supervision (1). Artificial intelligence is gaining prominence and has been studied in various fields of dentistry, including radiographic interpretation, caries diagnosis, orthodontic assessment, oral pathology and prosthodontic treatment planning. Because of the dependence of endodontic management on radiographic imaging, decision making and treatment planning, these disciplines are particularly amenable to AI adoption (2–5).

The purpose of this paper is to review contemporary developments in the application and effectiveness of advanced AI techniques in clinical endodontics as well as the potential uses of AI for augmenting information in clinical decision-making related to diagnosis, predicting complexity, standardizing diagnostic criteria and case based learning. It is also used in education: generating exam material (4), personalization of learning plans (5) and virtual simulation for exposure to preclinical and clinical endodontics (6). As LLMs like ChatGPT become increasingly available, AI chatbots are entering the educational ecosystem to interact with students on-demand and provide real-time feedback while also assisting faculty in content generation (2).

However, although these are promising developments, considerable gaps exist; AI in dentistry has been investigated internationally in systematic reviews; however, only limited evidence syntheses exist that explore the use of AI in endodontic education (6, 7). At present, evidence is mixed: studies vary in terms of methods employed, the AI platform and outcomes evaluated. Consequently, there is no consensus on a baseline understanding of what role AI plays in endodontics education, what benefit it provides and what obstacles or challenges remain (8–10).

Thus, the objective of this systematic review is to collect and summarize data regarding AI applications in endodontic education. This review summarises the current literature to inform educators, researchers and policymakers about whether AI has a role in future endodontic education and suggest gaps in knowledge where further investigation is warranted.

## 2 Materials and methods

### 2.1 Study design

This systematic review was performed according to the Preferred Reporting Items for Systematic Reviews and Meta-Analyses (PRISMA) 2020 guideline. The protocol was designed to include all pertinent studies published on AI, ML, DL, AR, VR and LLMs for use in endodontic education. For the current review, VR and AR have been included only when these technologies were evaluated as digitally enhanced educational technologies in which they were AI-adjacent (combined with interactive feedback or a simulation) or image-guided learning against an automatic assessment in endodontic training. They serve as an indication to develop a digital learning environment around AI-supported endodontic education.

### 2.2 Eligibility criteria

Eligible studies were defined by the following criteria:

- Population: Dental students (undergraduate, postgraduate); interns; residents; practitioners or patients participated in endodontic education.
- Intervention: Studies AI-based tools, ML/DL algorithms, VR/AR modules or LLMs in didactic or clinical training.
- Comparator: Conventional teaching style (lectures, textbooks, manual training, unguided diagnosis) or no intervention.
- Outcomes: Measurement of learning, diagnostic-accuracy, clinical skills, understanding by patients or perception on AI.
- Study type: RCT, quasi-experimental, cross-sectional, observational, systematic review and clinical validation.
- Exclusion criteria: Non-English publications, editorials, expert opinions, conference abstracts, and studies that did not examine endodontic education or the application of AI only in other dental specialties without an endodontic focus.

### 2.3 Information sources and search strategy

A comprehensive electronic search was performed in these databases:

- PubMed/MEDLINE
- Scopus
- Web of Science
- Cochrane Library
- Google Scholar (supplementary)

The search covered literature published from January 2015 to September 2025. Controlled vocabulary (MeSH terms) and free-text keywords were combined using Boolean operators.

The search was last updated in September 2025 to ensure inclusion of the most recent publications.

Search strategy: The search strategy was tailored for each database by using MeSH terms, keywords and boolean operators. The searches were performed independently by two reviewers. In the screening for Google Scholar, only 100 results at most were checked per search by relevance to minimize duplication and non-specific retrieval. The last search was performed on 15 September 2025. This step could potentially introduce language bias, since only studies published in English were included.

### 2.4 Study selection

Duplicates were removed using EndNote for all search results. Two reviewers screened titles and abstracts independently. Potentially relevant articles have full texts retrieved and reviewed against the eligibility criteria. Any inconsistencies were resolved by discussion with a third reviewer.

### 2.5 Data extraction

Data were abstracted using a standard form comprising the following:

- Study Characteristics (Author/Year/Country/Journal)
- Study design and sample population
- AI domain (ML, DL, VR, AR, LLM and hybrid).
- Type of application (didactic, preclinical, clinical, patient education)
- Comparator used (if any)
- Primary outcomes (i.e., diagnostic accuracy, exam scores, skill acquisition, comprehension, anxiety levels, satisfaction)
- Key findings and limitations
- Data were extracted independently by two reviewers and verified.
- Assessment of the risk of bias and quality
- Depending on the study design, appraisal tools appropriate for qualitative studies were used to assess the quality of included studies:
- RCTs: Cochrane Risk of Bias 2 (RoB 2) tool.
- Non-randomized studies: ROBINS-I tool.
- Cross-sectional studies: AXIS tool.
- Systematic reviews: AMSTAR-2 checklist.

Quality was evaluated by two reviewers, with disagreements resolved by consensus.

### 2.6 Data synthesis

A narrative synthesis was largely performed because of the heterogeneity in AI domains, educational outcomes and study designs. This included quantitative data e.g., diagnostic accuracy, sensitivity, specificity, exam performance and Likert-scale feedback were summarised (see Tables 1–4). Due to methodological diversity and outcome variability a meta-analysis was not attempted.

**Table 1:** Characteristics of included studies (2024–2025 updates highlighted).

S. No	Author (year)	Country	Study design	AI domain	Application	Sample/Setting	Key findings
1.	Aminoshariae et al. (2024) (11)	USA	Scoping Review	AI, VR/AR, LLMs	Endodontic education mapping	35 studies	Identified didactic/clinical uses, future gaps
2.	Alsalleeh et al. (2024) (12)	KSA	Experimental	AR	Root canal anatomy teaching	Dental undergraduates	AR improved efficiency and knowledge
3.	Karatekin et al. (2024) (13)	Turkey	Experimental	AR	Guided access cavity	3D-printed teeth	Improved usability and training outcomes
4.	Boztuna et al. (2024) (14)	Turkey	Deep Learning	ML/DL	Periapical lesion detection	Panoramic radiographs	High segmentation accuracy
5.	Deshpande et al. (2024) (15)	India	Systematic Review	VR	Endodontic training	12 studies	VR improved psychomotor skills
6.	Durmazpinar and Ekmekci (2025) (16)	Turkey	Comparative	LLM (ChatGPT-4o)	Endodontic case solving	60 cases	AI outperformed students (83% vs. 71%)
7.	Jalali et al. (2025) (17)	USA	Comparative	LLMs (7 chatbots)	Endo board questions	200 items	Variable accuracy; ChatGPT > Gemini
8.	Pul et al. (2025) (18)	Germany	RCT	AI assistance	Radiolucency detection	30 dentists, 50 radiographs	Reduced false positives; junior benefit
9.	Islam (2025) (2)	Pakistan	Cross-sectional	AI education	Patient information	100 patients	Improved comprehension, reduced anxiety
10.	Ma et al. (2025) (4)	China	RCT	LLM (GPT-4)	Exam generation	126 students	Greater coverage, lower discrimination
11.	Javed et al. (2025) (19)	Pakistan	Systematic review	VR-based endodontic learning	Undergraduate dental education studies	VR simulation was associated with improved learning experience, procedural confidence, and psychomotor training outcomes.	Systematic review
12.	Hu et al. (2025) (20)	China	RCT	VR simulation	Regenerative endodontics	120 students	Improved learning outcomes, satisfaction
13.	Ibraheem et al. (2025) (21)	Egypt	Comparative	AI detection	Caries/endo lesions	50 cases	Improved detection accuracy
14.	Szabó et al. (2025) (22)	Hungary	Clinical validation	DL	Lesion detection	Clinical radiographs	Assisted radiographic evaluation
15.	Kuru et al. (2025) (1)	Turkey	Comparative	LLMs	Dental trauma education	30 Qs × 5 models	Variable accuracy; ChatGPT superior

**Table 2:** Didactic vs. clinical applications of AI in endodontics.

Application	Didactic use	Clinical/Preclinical use	Examples
Radiograph interpretation	Training in reading PRs	AI-assisted diagnosis	Pul et al. (18), Ibraheem et al. (21)
Case diagnosis	Chatbot-based Q&A	Real-time support	Jalali et al. (17), Durmazpinar and Ekmekci (16)
Assessment tools	AI-generated exams, MCQs	—	Ma et al. (4)
Preclinical training	AR/VR modules for anatomy, access	Simulation of procedures	Alsalleeh et al. (13), Javed et al. (19), Hu et al. (20)
Patient education	ChatGPT explanations	Anxiety reduction, comprehension	Islam (2)
Robotics/Automation	Early-stage pilot	AR-assisted cavity access	Karatekin

**Table 3:** Quantitative outcomes from AI integration.

Study	Task	Comparator	AI outcome	Statistical result
Pul et al. (18)	Radiolucency detection	Unaided dentists	Accuracy 93.3% vs. 91.6%; false positives halved	*p* < 0.001
Durmazpinar and Ekmekci (16)	Case solving	Dental students	AI 83% vs. Students 71% accuracy	*p* < 0.05
Ma et al. (4)	Exam generation	AI vs. human exams	Coverage 81% vs. 72%; lower discrimination (0.35 vs. 0.49)	*p* = 0.027
Islam (2)	Patient comprehension	AI vs. leaflets	Comprehension +1.2; anxiety −0.9 (Likert)	*p* < 0.01
Hu et al. (20)	VR regenerative module	Control	Higher exam scores, satisfaction	*p* < 0.05

**Table 4:** Impact of AI on educational outcomes.

Domain	Outcome	Effect of AI	Evidence
Diagnostic training	Accuracy, false positives	Improved performance, esp. juniors	Pul et al. (18)
Didactic assessment	Exam coverage/discrimination	Higher coverage, lower discrimination	Ma et al. (4)
Case-solving	Decision-making accuracy	AI > students	Durmazpinar and Ekmekci (16)
Preclinical skills	Psychomotor accuracy, confidence	Significant improvement	Hu et al. (20)
Patient education	Comprehension, anxiety	Better comprehension, reduced anxiety	Islam (2)
Curriculum integration	Reliability of LLMs	Variable, requires supervision	Jalali et al. (17), Kuru et al. (1)

These reviews were included to provide an overview of current AI and digital educational applications in endodontics. They were not aggregated with primary studies for quantitative interpretation. Narrative synthesis was completed where there was overlap between the reviews and primary studies, in order to prevent duplication of evidence. The manuscript cites 26 references; however, 15 studies met inclusion criteria for qualitative synthesis (the remaining references were used to support the background, rationale and contextual discussion). References external to the final included-study set were not analysed as evidence for qualitative synthesis, and were instead cited where they offered contextual or background information.

The initial search identified 1,300 records from electronic databases and supplementary sources. After removal of 250 duplicate records, 1,050 records were screened by title and abstract. Of these, 900 records were excluded because they did not meet the inclusion criteria. A total of 150 reports were sought for full-text retrieval and assessed for eligibility. During full-text screening, 135 reports were excluded. Finally, 15 studies were included in the qualitative synthesis. Meta-analysis was not attempted given methodological diversity and outcome variability.

## 3 Results

### 3.1 Study selection

The first search resulted in 1,300 records from electronic databases and additional sources. Following removal of 250 duplicates, 1,050 records were screened by title and abstract. Nine hundred records were excluded of these because they did not meet the inclusion criteria.

This resulted in 150 reports were retrieved for full-text assessment. At this stage, 135 reports were excluded during full-text screening. Reasons for study exclusion included lack of relevance to endodontics, lack of educational or training focus, AI applications not relevant for endodontic learning, opinion-based publication, conference abstract only publication, duplicate thematic reports, insufficient data, or reporting limitations.

**Figure 1:**
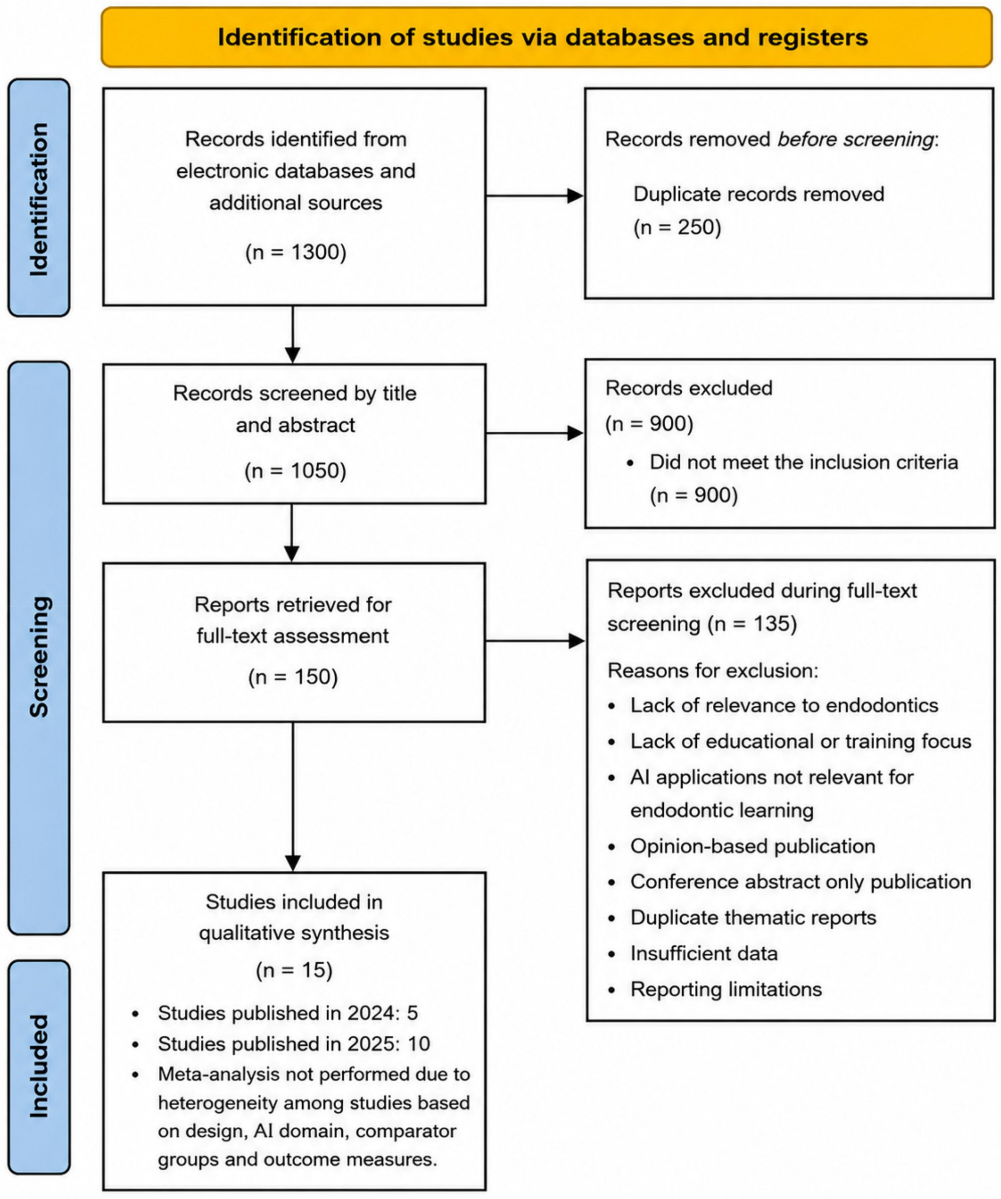
PRISMA flowchart.

In total, 15 studies were included in the qualitative synthesis. During these years, 5 studies were published in 2024 and 10 in 2025. Meta-analysis was not possible due to heterogeneity among studies based on design, AI domain, comparator groups and outcome measures (Figure 1).

### 3.2 Characteristics of included studies

The final qualitative synthesis included 15 studies published between 2024 and 2025. The types of AI applications assessed in these studies were augmented reality, virtual reality, large language models, deep learning or AI-assisted radiographic interpretation, patient education and endodontic case-based learning.

The included studies were heterogenous across AI topic. Studies on augmented reality mainly were related to learning root canal anatomy and guided access cavity preparation, while virtual reality and virtual simulation studies focused on preclinical skills development and procedural skill development. Studies analyzed the power of AI systems in solving endodontic cases, generating board-style questions, and dental trauma education and examinations using large language models. Radiographic detection of periapical lesions and related dental conditions was the topic of investigation in some deep learning and AI-assisted studies. Table 1 shows the leading characteristics of included studies.

### 3.3 Didactic vs. clinical applications

We categorized AI applications into didactic (exam generation, chatbot-based learning, patient education modules) and clinical/preclinical areas (AR/VR simulation, diagnostic support, lesion detection). Recent evidence demonstrates that LLMs (ChatGPT-4o, Gemini, Copilot) are testing students and platforms for Q&A; however, VR/AR is primarily examined in the preclinical endodontic training setting.

Endodontically, the clinical vs. didactic application of AI in education is summarized in Table 2.

### 3.4 Quantitative outcomes of AI integration

Measurable performance indicators were reported among RCTs and comparative studies. AI-assisted systems improved lesion detection accuracy and reduced false-positive interpretations compared with unaided assessment. In contrast, AI-generated exams covered much more content than faculty-generated and exhibited lower discriminatory ability compared with faculty-generated examinations.

Table 3 summarizes the quantitative effects of AI integration.

### 3.5 Educational impact

AI integration had a positive impact on students’ diagnostic ability, psychomotor skills in clinical exercises, examination performance and patient-centred communication. Results showed variability of chatbot reliability per platform, necessitating structured faculty oversight and curriculum incorporation.

Table 4 Educational outcomes of AI applications.

### 3.6 Risk of bias assessment

Several literature databases were searched to identify relevant studies, which had variable methodological quality (as assessed by risk-of-bias assessment). Interventions were assessed for risk of bias, with randomized controlled trials generally receiving a low to moderate risk, where concern was raised mostly about allocation concealment, participant blinding and outcome assessor blinding. Experimental and comparative studies had relatively high concerns, mostly because of non-randomized allocation, insufficient adjustments for confounding and differences in comparator populations. Cross-sectional studies showed moderate risk, mainly because of sampling issues and use of subjective or perception-based outcomes. Clinical validation studies had moderate concern as these were mostly based on curated radiographic datasets with very little external validation. The domains addressed by the integrated systematic and scoping reviews were generally relevant, but almost all lacked protocol registration, detailed publication bias assessment or formal grading of certainty-of-evidence.

**Figure 2:**
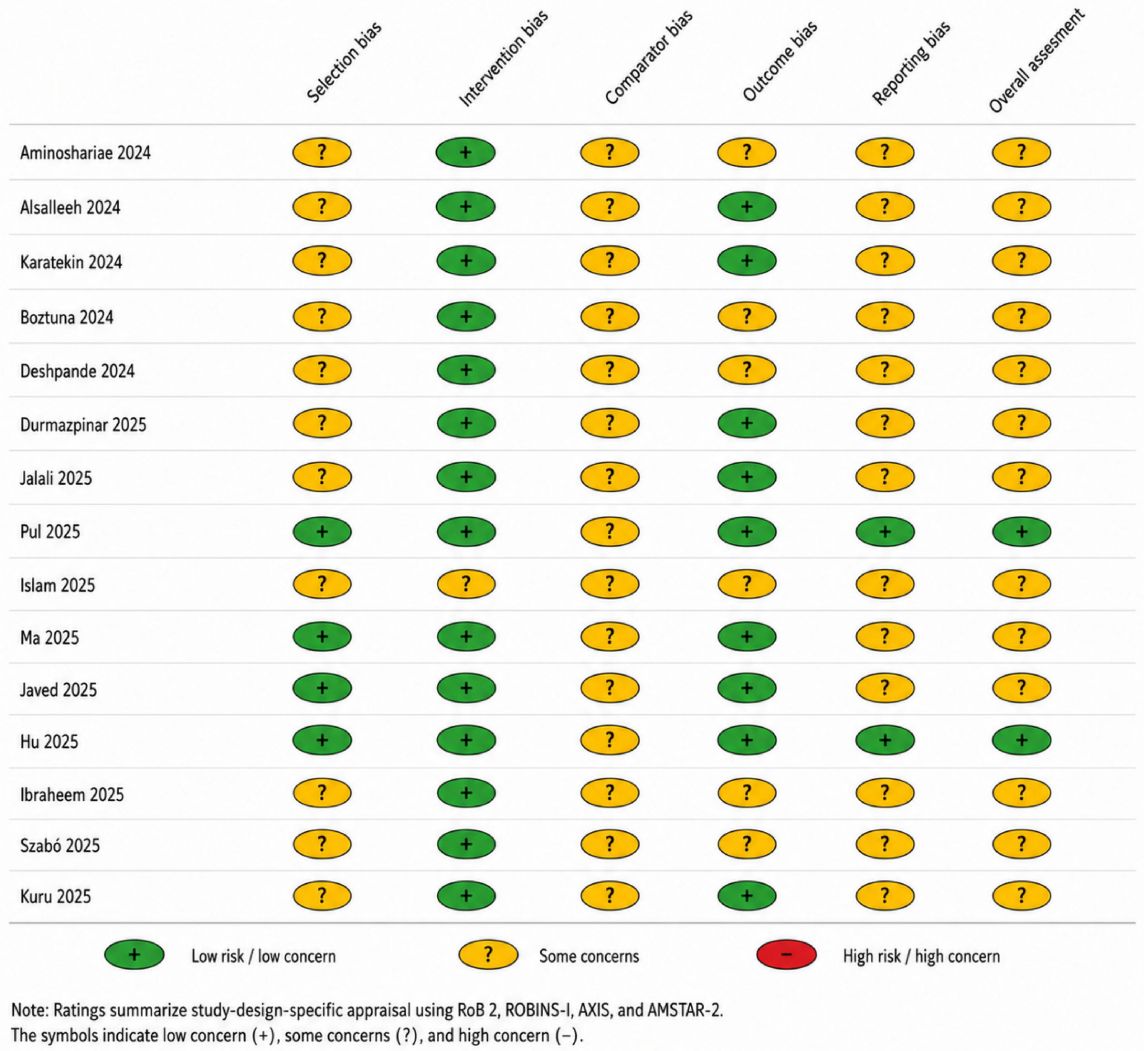
Traffic-light plot showing risk-of-bias assessment of included studies.

Reporting quality: Evaluation results were overall of moderate to high quality (Table 5, Figure 2).

**Table 5:** Summary of risk-of-bias assessment of included studies.

Study category	No. of studies	Tool used	Overall judgment
Randomized controlled trials	3	RoB 2	Low to moderate risk
Experimental educational studies	2	ROBINS-I	Moderate risk
Comparative studies	4	ROBINS-I	Moderate risk
Cross-sectional study	1	AXIS	Moderate risk
Clinical validation/diagnostic AI studies	2	ROBINS-I	Moderate risk
Scoping/systematic reviews	3	AMSTAR-2	Moderate quality

Total = 15.

## 4 Discussion

This systematic review summarizes current evidence regarding the role of AI in endodontic education up to 2025. AIs are emerging not just in diagnostic and clinical but didactic and simulation-based pedagogy, with promise yet warnings.

### 4.1 AI in endodontic education—benefits and advantages

The studies presented generally indicate that AI can improve diagnostic accuracy in endodontics. Example: AI-based interpretation of periapical lesions decreased false-positive diagnosis among diagnostically inexperienced clinicians. By contrast, in only a few comparative studies including endodontic case scenarios AI responses were more correct than the students’ response but these data come from small sample and should be understood as evidence of little superiority of the AI. The tangible trend of endodontic artificial intelligence is the capability of detecting periapical lesions (16), root fractures (17) and many anatomical structures with very high accuracy via convolutional neural networks (CNN) on cone beam computed tomography (CBCT) or periapical images (11).

In Education, VR/AR modules were more effective than traditional methods in psychomotor and preclinical skills. Virtual simulation evidence and systematic review findings, for instance, support VR-based endodontic treatment training increasing procedural competence and strengthening learner confidence compared to traditional methods. AR modules created for dental pulp (or root canal anatomy) also showed comparable effectiveness with increased student efficiency and performance in (12, 19, 20).

Another emerging frontier is the didactic use of LLMs. The experiments comparing AI- and human-generated exams reveal that AI produced broader content coverage but lower discrimination of high-performing students from less able ones. Also, informal assessment of other clinical chatbot platforms for endodontic Q&A raise questions regarding the reliability of these models since they evaluated a much smaller number of cases than in this study and generally did worse in testing more nuanced clinical situations compared to simple ones (4). Other chatbots in regenerative endodontics had a negative association between response accuracy and complexity of each query (16).

In addition, this paper presents evidence of AI in patient education from a large cross-sectional study showing that AI-generated educational output improves the learning experience to a much greater extent than traditional material by clarifying and limiting anxiety more effectively. For example, patient-facing applications are useful in increasing self-directed learning among dental students because content can be tailored to develop communication skills (2).

### 4.2 Challenges, limitations, and gaps

While there has been some progress concerning AI, there are still a number of existing limitations that can hinder widespread adoption in education. The first challenge is model transparency and interpretability—many AI or deeper models are black-boxes which make it impossible for educators to explain errors, or outputs that can result in unexpected results. Such an approach would be particularly problematic in an educational setting, where explainability is a key requirement. One other limitation was that the review protocol was not prospectively registered and, therefore, there may be more limited transparency in prespecified eligibility criteria and analytical decisions.

Second, dataset bias and limited dataset diversity remain persistent concerns. Given that many models are trained on homogenous datasets (e.g., specific populations, environments, machine settings, and even imaging protocols), they are likely to underperform in other real-world scenarios (11, 23) Most of the included studies were not externally validated through institutions or demographic cohorts.

Third, the inconsistency in reliability of LLM/chatbot platforms shows a great need for regulation. In addressing the questions related to complex endodontic conditions, some chatbots performed poorly. Uncurated such tools can spread misinformation and mislead the learners (17, 23).

Fourth, across studies there is inconsistency in measures of outcomes. Some use accuracy, sensitivity or specificity; others a Likert-satisfaction or confidence measure and yet more qualitative feedback. Yet, this heterogeneity limits comparability and prevents meta-analysis.

The fifth problem involves ethical, legal and pedagogical dilemmas. They include (but are not limited to) data privacy, especially for patient images and student performance data; how AI might de-skill students if overused; and the likelihood of transforming faculty use from content transmitters to facilitators. These worries are reflective of broader critiques sometimes found in the literature on AI in dental education (24).

However, there was significant heterogeneity restricting a direct comparison of study design, AI platforms and learning outcomes. The majority of included studies were single-center and relatively small. By restricting the publications to English language only, a selection bias could also have been introduced.

Last, often faculty awareness and preparedness is an issue. For AI, the survey also revealed significant gaps in knowledge, awareness and acceptance among dental faculty to this new technology; Successful integration of AI into dental education will require faculty development programs, institutional policies, and appropriate curriculum design (25), so not necessarily also an AI system. However, they were kept because the reviewed literature often addresses these methods simultaneously with AI-enabled simulation, image-guided learning and digitally augmented endodontic education. In the future, we recommend that reviews distinguish between AI-specific and broader digital education technologies. Endodontic education should place high particular caution and attention because responses may be influenced by these limitations of large language models, such as hallucination, prompt sensitivity, non-traceability to source(s), and reproducibility variability. These limitations are relevant for clinical education since they can unintentionally bias student reasoning by providing incorrect or unsupported explanations. LLM-generated content should not be relied upon as a standalone syllabus for teaching, and that faculty review is essential prior to integrating information distilled from LLMs in assessment, case discussion or patient education.

### 4.3 Integration and future directions

Several important strategies may support successful implementation. First, there are hybrid models that use human oversight of the AI so that the risks can be mitigated—e.g., assignments giving students feedback generated by the AI can be checked by instructors to confirm academic legitimacy. Second, validation studies at the levels of several institutions and with large sample sizes are required to ascertain generalization and robustness. Third, we may create XAI modules useful for students and teachers to provide transparency and trust (6). Fourth, standardization of outcome measures would make it easier over time to pool data for meta-analytic analysis and hence strengthen the evidence base for such reviews.

Curriculum design also needs to catch up: Students should learn about AI and Model limitations, model interpretation, ethics as part of their training. This is where faculty workshops and support services can fill in that adoption gap. However, robotic endodontic assistance and adaptive simulation systems and generative scenario creation (LLMs auto-generating case sim) are still new areas. In fact, there is groundwork on medical simulation showing that LLMs can generate prototype scenarios from semi-structured data (7, 26).

In summary, AI in endodontic education is at an inflection point. Thus, early-stage evidence supports possible diagnostic and simulation uses of the technology, however available evidence is small and heterogeneous reporting methods with varying outcome measures.

## 5 Conclusion

Employing a systematic review methodology, and extending the date of the literature search to be inclusive until end December 2025 for timeliness of reporting bias of emerging topics over time in this one field, this paper attempts a robust evidence synthesis on incorporating integration within education for AI with substantial capabilities demonstrated where specific cases such as diagnostics anticipate greater impact alongside simulation training and didactic tools for example. E.g., some studies made advancements in the accuracy and reduced error rates of diagnostic systems for detecting radiographic lesions, which are especially important when applied to novice learners. Psychomotor and procedural training can also be enhanced.

Large language models are promising for creating assessments and Q&A like features but their consistency is more sporadic in complex cases. Patient education applications further expand the educational role of AI. These strengths, however, come along with key challenges including lack of explainability, dataset biases, heterogeneous outcome reporting and ethical considerations as well as faculty readiness. Meanwhile, future work should focus on validated explainable AI models with instructor supervision and metrics of evaluation that have been previous met. When thoughtfully applied, AI has the potential to reinforce endodontic teaching and learning in the future.

## Data Availability

The original contributions presented in the study are included in the article/Supplementary Material, further inquiries can be directed to the corresponding author.
